# The acceptance of zinc biofortified rice in Latin America: A consumer sensory study and grain quality characterization

**DOI:** 10.1371/journal.pone.0242202

**Published:** 2020-11-11

**Authors:** Bo-Jane Woods, Sonia Gallego-Castillo, Elise F. Talsma, Daniel Álvarez

**Affiliations:** 1 Division of Human Nutrition and Health, Wageningen University and Research, Wageningen, The Netherlands; 2 HarvestPlus, c/o The Alliance of Bioversity International and the International Center for Tropical Agriculture (CIAT), Cali, Colombia; Indian Institute of Food Processing Technology (IIFPT), INDIA

## Abstract

Zinc deficiency is a major public health problem in vulnerable populations of Latin America and the Caribbean. Biofortification of rice (*Oryza sativa* L.) with zinc has the potential to alleviate zinc deficiencies. However, as plant breeding processes can alter grain culinary quality and favorable sensory attributes, grain quality and consumer acceptability need to be assessed prior to releasing a variety to the public. A grain quality characterization and a sensory acceptability analysis were carried out with two varieties of zinc biofortified rice and a local control both in Bolivia and Colombia. The aim of this study was to evaluate the physicochemical parameters that are significant in consumer acceptance and to determine the acceptability of zinc biofortified rice by consumers. Results of physicochemical parameters were analyzed using ANOVA. The sensory acceptability was evaluated in 243 adults utilizing a 7-point hedonic scale and a Wilcoxon’s signed rank test was used to determine the overall acceptability of the varieties. Biofortified rice variety T2-11 and MAC-18 -control 1- were equally accepted by consumers in Bolivia with no significant differences (p<0.05). The grain quality analysis reported that both presented long and slender rice grains (L>7.5 mm and L/B>3), an intermediate to high amylose content (>25%) and a similar level of chalkiness. In Colombia, the biofortified variety 035 presented a higher score in overall acceptance in comparison to biofortified variety 021 and the local variety CICA4 -control 2-. However, no significant differences were observed (p<0.05). Conversely to the other two varieties, the biofortified variety 035 presented the largest size grain (L/B = 2.97), a lower chalkiness and an amylose content above 25%. This study shows that the grain quality properties of rice have an influence on acceptability and that zinc biofortified rice varieties are accepted by consumers.

## Introduction

Hidden hunger, referring to a deficiency in essential micronutrients, is a major public health problem that is estimated to affect more than 2 billion people worldwide, especially those that live in low- and middle-income countries (LMICs) and with low socioeconomic status (SES) [1, 2]. Women and children below the age of 5 years are the two most vulnerable populations [3]. The main micronutrients deficiencies worldwide include zinc, iron, iodine, vitamin A and folate [4]. Zinc deficiency affects one third of the world’s population, and it is associated with a higher mortality in children [5–7]. It is linked with various health implications including growth retardation, impaired immune response and an increased risk of contracting infectious diseases such as diarrhea and pneumonia [6–8].

In Latin America and the Caribbean (LAC) there is a high prevalence of zinc deficiency, especially in children under 6 years of age and women 12 to 49 year of age [9–11]. Among others, Bolivia and Colombia are countries with a high degree of deficiency in this micronutrient [12]. This is particularly evident in populations that are situated in poor and rural regions [9, 13]. It is estimated that the prevalence of zinc deficiency is 61% in children under 5 years in rural Bolivia, raising up to 87% in certain regions, and 32% in urban children under 3 years [13, 14]. In Colombia, 36% of children under the age of 4 were zinc deficient and that the Atlantic (41%) and Amazonia (40%) regions had the highest prevalence [15]. In both countries, geographic location and poverty were strongly associated with an increased risk of zinc deficiency [16, 17].

Several strategies currently exist to combat “hidden hunger” including food fortification, distribution of dietary supplements and promotion of dietary diversification [5, 18]. However, in many situations these methods are unsuccessful due to elevated costs, complex logistics, low compliance and limited access to a variety of food [19, 20]. Biofortification through conventional breeding is a relatively new food-based intervention targeted at LMICs that entails the process of increasing the micronutrient content of staple foods through cross breeding of specific crop varieties, by genetic engineering or the application of fertilizers [21]. Unlike alternative approaches, biofortification enhances the micronutrient content in the crop during the growth phase [5]. It comprises an initial investment for developing a variety which constitutes a part of the daily diet of the target population, that can be grown and consumed yearly, with no additional production costs compared to a commercial variety [18, 22]. This establishes the opportunity of increasing the daily micronutrient intake [19, 23]. Additionally, surplus production can be marketed thus impacting the household income [24]. It is a cost-effective and sustainable strategy of supplying micronutrient-rich staple crops to rural communities and vulnerable populations who have limited access to other existing nutritional interventions [18, 25, 26].

Rice (*Oryza sativa* L.) is one of the most consumed cereals worldwide, and it is considered a staple food by 50% of the world’s population [8, 27]. In Bolivia and Colombia, rice is the cereal with the highest consumption nationwide [15, 28]. Unpolished rice contains a concentration of zinc between 15 and 58 mg/kg [29]. However, it is important to note that an estimated 22% of the zinc content is lost during post-harvest processes caused mostly by mechanical treatments such as milling and cooking [30, 31]. Furthermore, due to rising levels of carbon dioxide as a result of climate change, the zinc content of grains, legumes and tubers is continuously decreasing, potentially placing an estimated 140 million more people at risk of zinc deficiency by 2050 [32]. The release of rice varieties that have been biofortified with a high zinc content could combat and prevent increasing rates of deficiency of this micronutrient, particularly when combined with dietary diversity and nutritional education [18, 33, 34].

The process of zinc rice biofortification through conventional breeding comprises several steps. The first step is the identification of rice varieties that accumulate high zinc levels from the global germplasm banks. Next, parent building is implemented to obtain varieties with enhanced zinc levels, high agronomic performance, and consumer quality traits. The last step comprises crossings with local varieties to obtain a final product. This final product must fulfill requirements such as meeting the preferences of local producers and consumers, be high yielding, and present enhanced zinc levels that are stable across sites and generations. The promising lines are then subjected to national performance trials as a mandatory governmental requirement prior to a subsequent release to the public [29].

The impact of zinc biofortified rice on human nutritional status and the efficacy of the biofortified variety is dependent on its bioavailability. This parameter is defined as the amount of nutrient that is absorbed and utilized through normal metabolic pathways in the body [35]. It is strongly influenced by components that promote or hinder mineral absorption such as phytic acid (PA), which is naturally present in rice [36–38]. Thus, it is advised to examine the phytic acid to zinc (PA:Zn) molar ratio of the rice to estimate zinc absorption [39]. Despite the factors that limit the zinc levels in rice, biofortified rice varieties can provide more zinc than local varieties when consumed as the main staple. Therefore, they have higher potential to contribute to the Estimated Average Requirement (EAR) and improve human zinc status [10, 40, 41].

Although studies investigating the acceptance of nutritionally enhanced rice are limited, the majority report findings with an overall liking for the control, with one study in Cuba favoring the zinc and iron enhanced variety [42–44]. Research has shown that the success of biofortification depends on the acceptance of the newly bred crop varieties by consumers, among other aspects [20]. Thus, in advance of releasing a variety, it is necessary to consider parameters important to the consumer that may influence the outcome.

Consumer acceptability studies have reported that grain quality of the rice is of high importance for consumers and although quality characteristics could vary across countries and regions, some are widely shared [45]. Grain quality is defined by physical characteristics including grain size and appearance, cooking parameters and sensory attributes [46–48].

Although measures in the crop development phase of rice varieties are taken to ensure that the sensory qualities of the varieties resemble those of existing in commercial varieties, biofortification can cause slight changes in sensory and physical attributes [19]. Therefore, the chances of acceptance are increased when the attributes and quality of the original favored grain are mirrored or reproduced in the new biofortified variety [20, 45]. Consequently, grain quality is a focal point in crop breeding stages, which facilitates the generation of crops that resemble favorable attributes of the locally consumed variety [5]. Prior to selecting a superior variety for potential release, it is therefore strongly advisable to establish acceptance at consumer level to predict consumer liking and in turn, increase odds for a successful acceptance [20, 49]. Hitherto, literature has revealed contrasted findings regarding the acceptability of zinc biofortified rice varieties [23, 42–44]. Besides, no grain quality analysis accompanied the sensory study to identify the key parameters critical for consumer acceptability. Thus, no certainty of consumer acceptability could be expected after the release of the rice varieties to the public.

The aim of this study was to evaluate the grain quality characteristics and the sensory acceptability of two biofortified and one local rice variety in both Bolivia and Colombia. The outcome of this work was considered as the final determinant step for the selection of the first biofortified rice varieties to be released in both countries.

## Materials and methods

### Rice samples

#### Grain characterization

In this study, two varieties of biofortified rice were compared against one local commercial variety in each of the two countries. The two biofortified varieties PCT-25-C2-329-4-2SR-5P (referred to as T2-10), CT22154-9P-1SR-1P-3SR (T2-11) and the local variety MAC-18 (control 1) were planted, harvested and milled in Santa Cruz Department, Bolivia between 2017–2018. The two biofortified rice varieties BF14AR021 (021), BF14AR035 (035) and the local variety CICA4 (control 2) were likewise planted, harvested and milled in Bolívar Department (north coast of Colombia) between 2019–2020. These regions hosted the required national agronomic trials that are mandatory prior to releasing the selected varieties to the public. The biofortified rice varieties were produced in the same environmental conditions (altitude, sea level, environmental conditions) and with the same agronomic management as the local varieties to obtain similar culinary and industrial characteristics. The control varieties used in this study were chosen based upon consumption acceptance in each region. The rice varieties used in this study were stored for 3 months prior to the sensory test.

Grain dimensions such as grain length, length-breadth ratio (L/B) and chalkiness of the endosperm from each line were analyzed using a Vibe QM3 image analyzer (Vibe Imaging Analytics Ltd., USA). This equipment was designed specifically to measure rice kernel characteristics and uses a high-resolution digital camera to capture the image to be analyzed. After capturing an image of rice kernels, image processing is performed using a software calibrated to measure, count and classify kernel size, shape and color [50]. For the analysis, 25 g of milled rice kernels were used. Amylose content of milled rice was measured by a NIRS System 6500, with a reflectance of 1/log R, and a 400–2498 nm wavelength range [51]. The whiteness was measured with a commercial Kett whiteness meter (Rice Milling Meter, Model MM1D, Satake Co., Japan). An automatic rice cooker (Model CKSTRC4711, Oster Electrics, USA) was used to determine the cooking time. To carry out the cooking the protocol described by Poritosh et al. [52] was followed with modifications by the FLAR Rice Quality Laboratory. To start, distilled water was added at a temperature of 25°C and at a ratio of rice to water of 1:2. Then, the cooking cycle was activated and heated at full power, so that the water reached and stayed at boiling point of 100°C. Once the rice absorbed all the water and the rice was complete cooked, the unit automatically switched to the "keep warm" cycle, keeping the rice at a safe temperature of approximately 65°C. This temperature prevented the rice from being overcooked and kept the rice warm until it ready to be served. The cooking time was registered from the moment the rice cooker was switched on until the time it automatically turned off.

#### Zinc and phytic acid analysis

An Oxford X-Supreme 8000 energy-dispersive X-ray fluorescence spectrometer (XRF) (Oxford Instruments, UK) was used to measure zinc concentration in the six rice samples, as described by Paltridge et al. [53]. To conduct the measurement, 5 g of rice grains were introduced in the sample cups, consisting in aluminum cups containing inner polypropylene cups. The cups were sealed with 4 μm Poly-4 XRF sample film and shaken gently to distribute uniformly the grains. Then, the cups were placed in the XRF instrument and scanned.

The phytic acid (PA) content was determined according to the Latta and Eskin [54] with modifications. For the analysis, 1 g ground sample was extracted with 20 mL of 0.65 M HCl for 2 h on a multivortex. The extract was centrifuged for 15 min at 3800 rpm and 5 mL of the supernatant was introduced into the Poly-Prep chromatography columns (Bio-Rad Laboratories, USA, 100–200 mesh AG-1-X8 chloride anion exchange resin) to separate the PA from the sample extract. The interfering compounds and inorganic phosphorus were removed with 5 mL of ultrapure water (18 MΩ) followed by elution with 10 mL of 0.07 M NaCl. The PA bounded to the resin was eluted with 30 mL of 0.7 M NaCl, and a 0.9 mL aliquot of the eluate was vortexed with 0.3 mL of Wade reagent (0.03% FeCl3 (III), 0.3% sulfosalicylic acid). The absorbance of the salicylate-Fe (III) complex was determined on a spectrophotometer (BioTek Instruments, Inc., USA, 500 nm). The PA concentration was calculated using a standard curve (0–60 mg/mL) that was obtained with phytic acid dipotassium salt (Sigma-Aldrich, Canada) [55].

#### Potential contribution to the daily zinc intake and Estimated Average Requirement (EAR)

To estimate the potential impact of these rice varieties on the nutritional zinc intake in children under 5 years and women of reproductive age, the contribution to the Estimated Average Requirement (EAR) was calculated, assuming a 30% bioavailability [41, 56]. Available rice consumption data was used when possible to calculate zinc intake. In Bolivia, there is no official consumption data for children of that group of age. Therefore, an average of the consumption data in South American children between 3–5 years old of 42 g per day was used [57]. In the case of Colombia, the rice consumption data for children under 5 years old was used, and it is 59 g per day [15]. Due to the absence of intake data for women in Bolivia, the intake data of rice per capita consumption utilized was 114 g per day, obtained from food balance sheets [28]. For Colombia, the consumption amount of 114 g per day for women in reproductive age was used [15]. In all cases, a 78% zinc retention after milling and cooking was considered [30, 31].

### Sensory evaluation

#### Consent and ethics statement

Informed written consent was obtained from willing participants before the start of the study in Bolivia and Colombia. Ethical approval was obtained from the Institutional Review Board (IRB) of The International Center for Tropical Agriculture (CIAT) with the corresponding IRB case numbers: 2019-IRB-19 and 2018-IRB-02. The study protocol was approved by all actors involved: in Bolivia by the Research Center for Tropical Agriculture Santa Cruz (CIAT-SCZ); and in Colombia by the partners of the University of Cordoba, Fedearroz, the Foundation of Canal del Dique Compas, and the National Learning Service in Cartagena (SENA).

#### Study areas

This study was conducted in Bolivia in the department of Santa Cruz, in the municipalities of Yapacaní, San Juan and General Agustín Saavedra at the CIAT-SCZ local experimental stations. The department of Santa Cruz is a populous urban region with over 1.6 million inhabitants (Population Stat, 2019). Although information on zinc deficiencies in Santa Cruz is sparse and thus may have a lower risk of deficiencies, it has been recommended due to its importance in agricultural production and distribution as a ‘breadbasket region’ in Bolivia. Thus, it has high potential for use as a base for providing biofortified crops to the rest of the country and potentially increases the chance of national acceptance of the new varieties [58]. The experimental stations of CIAT-SCZ were chosen as they are situated near the three recruitment locations in Bolivia, which have practical facilities and a population with low-middle SES.

In Colombia, the study was conducted in the department of Bolívar at the local campus of the public National Training Service (SENA) in Cartagena city. It is a municipality with more than 1 million inhabitants and is a port city situated in Colombia’s Caribbean coast with predominantly an urban population and a tropical savanna climate [59]. The Atlantic region in Colombia, which includes Cartagena, was selected due to high rates of zinc deficiency (41%) in infants aged 0–4 years [15]. SENA in Cartagena was chosen as the location to implement this sensory trial due its suitability for recruiting subjects from low to middle SES with a minimum of basic education and practical facilities.

The stations chosen in both countries corresponded to central locations as recommended to implement a sensory study [60]. Additionally, both study sites were chosen based on populations of low-middle SES, nutrient deficiency risk, high rice consumption rates and the presence of local partners. Furthermore, alongside agronomic activities such as growth, harvesting and storage of rice varieties, agronomic evaluation trials for the release of materials directed by HarvestPlus and CIAT are being carried out.

#### Study participants and recruitment

In this study, adults were eligible for inclusion if they met the following criteria: aged between 18–64 years, had at least a basic level education, lower-middle SES, willing to sign a consent form to participate, and resident in the study area with no existing serious diseases or allergies to rice. The sample size was determined according to Lawless and Heymann [60], which defined a number of a minimum of 75 consumers as sufficient for statistical analysis and test sensitivity [61, 62]. The criteria for participation was provided to our partners of CIAT-SCZ (Bolivia) and SENA Cartagena (Colombia) which assisted in the consumers recruitment. Both procedures were carried out utilizing invitation flyers and verbal communication within the chosen locations.

In Bolivia, 108 adults between the ages of 18–64 years were recruited at the municipalities of Yapacaní, San Juan and General Agustín Saavedra. Similarly, in Colombia, 180 participants between the ages 18–64 years from SENA Cartagena were recruited. Participants were recruited at the experimental stations of CIAT-SCZ (Bolivia) and SENA in Cartagena (Colombia).

The sensory trials were carried out during the week of 2nd - 4th October 2018 in Bolivia and the week of 30th October - 2nd November 2019 in Colombia. The baseline and sensory survey questionnaires were written and designed in the local language, Spanish [63].

#### Standardized preparation of rice samples

A pilot test was carried out in the premises of HarvestPlus at CIAT in Colombia to ensure optimum temperature and timing of rice cooking, and to set standardized preparation and cooking conditions for all rice varieties being tested. All rice varieties were prepared, cooked and served in the same manner. For each trial session, 1 kg of each rice variety was added to 2 L of water and 20 g of salt, boiled at a temperature of 90–95°C for 25 min and cooked for 20 min (a total time of 45 min). Three rice cookers of the brand “Black and Decker” were used–one per variety tested to standardize the preparation of each sample. Rice was kept warm in the rice cooker at a temperature of 65–75°C in between tastings. No alteration in attributes of the rice were expected in storing rice at this temperature for a period of 0–24 h [64].

All rice samples were measured out in 30 g portions using predetermined plastic measuring spoons of 1/8 oz into paper cups and placed on a serving tray. One of each of the three varieties to be sampled were then organized in random order with pre-assigned 3-digit code labels and served to the participant. Sample temperatures were served at a temperature of around 40–50°C as determined during the pilot test. In addition, participants were provided with a 4 oz cup of water to rinse their mouth before and in-between tasting different samples.

#### Acceptability study protocol

The same method was followed for the sensory acceptability study in both locations. Participants were explained the procedures involved in the study. The sensory analysis study included answering a questionnaire and tasting a sample of the three varieties of cooked rice. The same questionnaire was used in both Bolivia and Colombia except for question 5c in section 1. For Bolivia, the category ‘Farmer’ was listed as an option for occupation, whereas for Colombia this was replaced by ‘Student’ in accordance with the population that was expected in the study location (S1 File).

Once collected, participants were given the sensory questionnaire with an individual ID number to ensure confidentiality. The first section was to be answered before the start of the trial. It consisted of questions pertaining to baseline information such as age, frequency of rice consumption and decision-making when purchasing rice. Following this, participants were served a tray of three small rice samples in carton colorless cups (4 oz) each with randomized 3-digit code on a neutral serving tray. To avoid bias of samples, each sample cup set was arranged differently on the trays using predetermined stickers with numbers allocated at random. Samples were prepared using a double-blind method, so that neither the experimenters nor participants knew which variety was in which cup. Two of the researchers were responsible for labelling the samples according to the variety using a pre-determined system of numerical coding. This prevented allocation bias in the results of this research.

The second section of the questionnaire required participants to rate their liking of the sensory attributes (color, size, smell, taste and texture) of the three samples individually using a 7-point balanced hedonic scale utilizing faces and basic language to ensure understanding for untrained assessors. The hedonic scale is a useful tool to assess in an ordinal level the degree of liking for food products, assuming that consumer preferences can be categorized by responses based on like and dislike [60, 62]. Additionally, the face scale is particularly recommended in LMIC where a certain level of low literacy can be expected [60, 65]. The scale used in this study consisted of three degrees of disliking (1–3), one for neutral (4) and three degrees of liking (5–7) and it was developed following the protocol described by Lawless and Heymann [60] with modifications by Carrillo et al. [66].

Each participant participated for a total time of 40 min approximately. Sensory trials were carried out in a total of five groups over a period of two days, two in the morning and one in the afternoon. There was a maximum of 30 participants permitted per group.

### Statistical analysis

Statistical analysis for grain quality characteristics, and zinc and phytate content among rice samples by country was carried out utilizing the Minitab software (version 19.2020.1, Minitab Inc., USA). As data were normally distributed, a one-factor ANOVA test was used to show overall significant differences of groups. Tukey’s HSD post-hoc was used to test significant differences (*p*<0.05) among sample means pairwise. Mean and standard deviation were reported. Differing superscript letters reported significant differences between pairs (*p*<0.05).

Statistical analysis of the sensory data was carried out using IBM SPSS statistics 22 (version 23.0.0.2, ICM Corp. Released 2015). Considering that the data was not normally distributed, non-parametric tests were used. Medians and interquartile ranges were reported to show differences in overall hedonic scores of the rice varieties across study sites. Median liking was defined as the median value of all assessed attributes. Wilcoxon signed-rank tests were carried out to test the differences in population mean ranks between rice varieties (overall liking) within the two study sites. Differences and significance (*p*<0.05) were reported. Furthermore, as individual measures on 7-point hedonic scales do not provide information on relative differences, they are considered as ordinal data. Thus, Wilcoxon’s nonparametric test was used to determine whether there were significant differences between single attributes and rice varieties within study location. Median values were reported and differences between attributes were differentiated via superscript letters. Data from the individual scores of single attributes were treated as ordinal. Statistical significance was determined at a *p*<0.05. Participants with missing data on attributes were still included in the analysis but were not imputed as missing data recorded was <5%, therefore were considered to be missing at random.

## Results

### Grain quality

Table 1 lists the physicochemical characteristics of the rice samples from Bolivia and Colombia that were analyzed in this study. In Bolivia, control 1 was significantly larger than the biofortified varieties T2-10 and T2-11. In grain length and shape, measurements reported that all varieties were extra-long (L>7.5 mm) and slender (L/B>3.0) [51]. The variety T2-11 showed a similar level of chalkiness than control. However, the chalkiness level of the variety T2-10 was significantly higher than both the control 1 and T2-11. Kett degrees, a measure of whiteness, were slightly higher in both biofortified varieties compared to the control. The amylose content and cooking times were similar in all three varieties with no significant differences. In Colombia, both biofortified varieties presented significantly larger sizes and lower levels of chalkiness in comparison to control 2. The average length of variety 035 was greater than 6.5 mm, which corresponds to the ‘long grain’ category, whereas control 2 presented a grain length of less than 5.5 mm, which classifies it as a ‘short grain’ rice [51]. The level of chalkiness was significantly lower in the control when compared to the two biofortified varieties. However, Kett degrees were similar in all three. Control 2 presented an amylose percentage of less than 25% which was significantly lower in comparison to both biofortified varieties. Cooking time was similar in all three with no significant differences.

**Table 1:** Physicochemical characteristics for the rice varieties.

Country	Sample	Size (L/B)^a^	Chalkiness	Kett degrees (%)	Amylose content (%)	Cooking time (min)
Bolivia	Control 1	4.27 ± 0.04^a^	0.42 ± 0.03^b^	44.87 ± 0.45^c^	32.50 ± 0.60^a^	27.0 ± 1.0^a^
	T2-10	3.66 ± 0.10^b^	1.17 ± 0.15^a^	50.13 ± 0.64^a^	32.23 ± 0.42^a^	26.3 ± 1.5^a^
	T2-11	4.24 ± 0.05^a^	0.43 ± 0.06^b^	48.73 ± 0.15^b^	33.10 ± 0.53^a^	27.0 ± 1.0^a^
Colombia	Control 2	2.25 ± 0.14^b^	2.02 ± 0.18^a^	46.50 ± 0.20^a^	24.57 ± 0.97^b^	26.7 ± 0.6^a^
	021	2.76 ± 0.09^a^	1.33 ± 0.06^b^	46.23 ± 1.07^a^	31.87 ± 1.05^a^	27.0 ± 1.0^a^
	035	2.97 ± 0.06^a^	1.00 ± 0.40^b^	46.03 ± 1.16^a^	30.23 ± 0.93^a^	26.0 ± 0.6^a^

Values are presented as mean ± SD. Means with different superscript letters (a,b,c) in the same column by country are significantly different with Tukey’s test (*p*<0.05); n = 3 sample replicates.

^a^L = length and B = breadth in mm.

### Zinc and phytic acid content

Zinc and phytic acid (PA) content are shown in Table 2. The biofortified variety in Bolivia T2-11 presented significantly (*p*<0.05) higher zinc levels than control 1. Zinc levels of the biofortified variety T2-10 were higher than the control 1 and lower compared to the biofortified variety T2-11. Both were not significantly different (*p*>0.05). Phytic acid levels were significantly lower in control 1 in comparison to T2-11, and not significantly compared to the T2-10. Likewise, the differences between T2-10 and T2-11 were not significant. In Colombia, on the other hand, zinc levels were significantly lower in control 2 compared to both biofortified varieties 021 and 035. However, phytic acid levels were not significantly different. Differences were observed between the biofortified varieties, being the phytic acid levels in the variety 021 significantly higher than the variety 035.

**Table 2:** Zinc and phytic acid content for the rice varieties.

Country	Sample	Zinc (mg/kg)	Phytic Acid (mg/g)
Bolivia	Control 1	17.33 ± 2.42^b^	0.61 ± 0.02^b^
	T2-10	22.03 ± 0.64^ab^	0.86 ± 0.11^ab^
	T2-11	24.70 ± 0.78^a^	1.02 ± 0.02^a^
Colombia	Control 2	18.35 ± 2.05^b^	2.66 ± 0.08^ab^
	021	26.77 ± 1.00^a^	2.80 ± 0.00^a^
	035	26.57 ± 0.72^a^	2.45 ± 0.08^b^

Values are presented as mean ± SD. Means with different superscript letters (a,b) in the same column by country are significantly different with Tukey’s test (*p*<0.05); n = 3 sample replicates for zinc, except control 2 in Colombia where n = 2 sample replicates; n = 2 sample replicates for phytic acid.

An estimation of the PA:Zn molar ratio was made using the mean values of phytic acid and zinc of each variety presented in Table 2, and considering the molecular weights of phytic acid (660 g/mol) and zinc (65 g/mol) [67]. In Bolivia, the control 1 presented a PA:Zn molar ratio of 3.61, and the two biofortified varieties 4.20 (T2-10) and 4.53 (T2-11). In Colombia, the molar ratio was 13.69 for the Control 2, 11.79 for the biofortified variety 021 and 10.32 for the biofortified variety 035.

### Potential contribution to the daily zinc intake and EAR

The potential contribution of zinc to the Estimated Average Requirement (EAR) is presented in Table 3. The biofortified variety T2-11 in Bolivia is estimated to provide 18% of the EAR in children under 5 years and 22% in women, compared to 12% in children and 16% in women provided by the commercial variety. In Colombia, it is estimated that the biofortified variety 035 can provide up to 27% and 24% of the EAR in children under 5 years old and women, respectively, compared to the commercial variety, with a contribution of 18% and 16%, respectively.

**Table 3:** Daily zinc intake values and EAR estimate for the rice varieties per study site.

Population	Country	Sample	Zn (mg/kg)	Rice consumption^a^ (g/day)	Zn intake^b^ (mg)	Contribution to EAR^c^ (%)
Children	Bolivia	Control 1	17	42	0.6	12
		T2-10	22	42	0.7	16
		T2-11	25	42	0.8	18
	Colombia	Control 2	18	59	0.8	18
		021	27	59	1.2	27
		035	27	59	1.2	27
Women	Bolivia	Control 1	17	114	1.5	16
		T2-10	22	114	2.0	20
		T2-11	25	114	2.2	22
	Colombia	Control 2	18	114	1.6	16
		021	27	114	2.4	24
		035	27	114	2.4	24

^a^Children: Bolivia = 42 mg (South American average in children 3–5 y consumption [57]; no official data for Bolivia); Colombia = 59 mg (children 1–4 y consumption [15]). Women: Bolivia = 114 g (food balance sheets [28]); Colombia = 114 g (women 13–17 y consumption [15]).

^b^Zinc cooking retention = 78% [30, 31].

^c^Estimated Average Requirements (EAR) for zinc: children 4–6 y = 4.6 mg/d; women = 9.9 mg/d, assuming a 30% absorption efficiency [56].

### Sensory acceptability

Table 4 displays the descriptive characteristics of the participants in this study. Due to missing data (>5%) and non- an assessment of not compliance with the inclusion criteria, 45 participants were excluded from the final analysis, 11 in Bolivia and 34 in Colombia. The total study population included for analysis consisted of 243 adults aged 18–64 years old. In Bolivia out of the 97 participants, 38% were female and 62% male. Similarly, in Colombia, out of 146 participants, 45% were female and 55% male. The average age of participants in Bolivia in this study was 34 (±12.3) years old and in Colombia at 24 (±8.9) years old. Most of the study participants were educated above secondary level, 81.4% in Bolivia and 76.0% in Colombia. Regarding principal occupation, in Bolivia 49.5% of participants were employed, 20% were farmers, 10% were housewives and 20% selected ‘none’. In Colombia, 82.9% participants were students, 14% were employed and 27% selected ‘none’. In both locations, rice was mostly consumed daily: Bolivia 98% and in Colombia 92%, or weekly at 2% and 8% respectively.

**Table 4:** Descriptive characteristics of participants by study site.

Variables	Bolivia		Colombia	
	(n)	(n = 97)	(n)	(n = 146)
AGE		34 ± 12.3		24 ± 8.9
AGE GROUP (years)				
18–24	30	30.9	109	74.7
24–34	29	29.9	23	15.8
35–44	17	17.5	6	4.1
45–64	21	21.6	8	5.5
GENDER				
Female	37	38.1	65	44.8
Male	60	61.9	80	55.2
EDUCATION				
Primary	4	4.1	6	4.1
Secondary	14	14.1	29	29.9
Further education	79	81.4	111	76.0
OCCUPATION				
Employed	47	49.5	20	13.7
Housewife	10	10.5	1	0.7
Farmer/Student	19	20.0	121	82.9
None	19	20.0	4	27.0
FREQUENCY OF RICE CONSUMPTION				
Daily	94	97.9	134	92.4
Weekly/1-2 times a week	2	2.1	11	7.6

Values are presented as mean (± SD) for continuous variables and as percentages (%) for categorical variables.

In Table 5, median hedonic scores and interquartile ranges of the three rice varieties tested in Bolivia and Colombia are displayed alongside reported significant differences (*p*<0.05) in population mean ranks (overall liking) between rice varieties within the two study sites. In Bolivia, regarding overall liking, the varieties T2-11 and control 1 equally scored and were higher than T2-10. Significant differences were found between the overall liking of the two different rice varieties T2-11 and T2-10 (*p*<0.05) and between control 1 and T2-10. No significant differences (*p*>0.05) in average liking were found between control 1 and T2-11. In Colombia, results showed that on average, for overall liking across the three varieties, 035 scored higher than the other two varieties (control 2 and 021). Results showed that the differences between overall liking of all three pairs: control 2/021, control 2/035 and 035/021 were not significant (*p*<0.05).

**Table 5:** Comparison of overall hedonic liking scores per study site and rice variety.

Country	Rice sample	Overall hedonic liking scores^a^	Differences between varieties^b^
Bolivia	Control 1	5.8 (5.2, 6.4)	-2.31^a^
	T2-10	5.6 (5.0, 6.0)	-0.08^b^
	T2-11	5.8 (5.4, 6.4)	-2.71^a^
Colombia	Control 2	5.3 (4.2, 5.8)	-0.54^a^
	021	5.4 (4.4, 5.8)	-1.69^a^
	035	5.5 (4.6, 6.0)	-1.37^a^

^a^Values are presented as median differences in study samples across settings and are shown as medians and interquartile ranges (25, 75).

^b^Values with different superscript letters (a,b) are significantly different with Wilcoxon’s non-parametric z-test (*p*<0.05).

Median hedonic scores and interquartile ranges of individual sensory attributes (color, size, smell, taste, and texture) for each rice variety tested in the two study locations are displayed in Table 6. In Bolivia, reported median scores showed that participants on average scored the same in liking for all attributes in the three varieties (6.0), except for rice texture in variety T2-10, which was seemingly least rated presenting a lower median average score (5.0). However, significant differences (*p*<0.05) in the attribute size was found between varieties control 1 and T2-11 vs T2-10, which was the least rated. Similarly, there were significant differences (*p* = <0.05) in smell between varieties control 1 and T2-11 vs T2-10, which was the least scored. No other significant differences (*p*>0.05) were found between the median liking scores of attributes for the three varieties.

**Table 6:** Median hedonic scores of sensory attributes of the local control varieties and the biofortified varieties with comparisons across study sites.

Country	Variety	Color	Size	Smell	Taste	Texture
Bolivia	Control 1	6.0 (5, 6)^a^	6.0 (6, 7)^a^	6.0 (5, 7)^a^	6.0 (5, 7)^a^	6.0 (5, 7)^a^
	T2-10	6.0 (5, 6)^a^	6.0 (5, 6)^b^	6.0 (5, 6)^b^	6.0 (5, 6)^a^	5.0 (5, 6)^a^
	T2-11	6.0 (6, 7)^a^	6.0 (5, 6)^a^	6.0 (5, 6)^a^	6.0 (6, 7)^a^	6.0 (5, 6)^a^
Colombia	Control 2	6.0 (5, 6)^a^	5.0 (4, 6)^a^	6.0 (5, 6)^a^	6.0 (4, 6)^a^	5.0 (3, 6)^a^
	021	6.0 (6, 6)^b^	6.0 (5, 6)^a^	5.0 (4, 6)^b^	5.0 (4, 6)^a^	5.0 (3.7, 6)^a^
	035	6.0 (6, 6)^b^	6.0 (5, 6)^a^	6.0 (5, 6)^a^	5.0 (4, 6)^a^	5.0 (3.7, 6)^a^

Values are presented as median values of individual attributes per rice variety and interquartile ranges (25, 75). Medians per column by attribute with a different superscript letter (a,b) significantly differ using Wilcoxon’s non-parametric z- test (*p*<0.05).

In Colombia, slight variations in median hedonic scores for overall liking of attributes in all three rice varieties were observed. Color scored equally for all varieties (6.0), and significant differences were found between the overall liking of color in control 2 and 035/021 varieties. Size was rated higher in 035 and 021 (6.0) compared to control 2 (5.0) but with no significant differences (*p*>0.05). Rice variety 021 was significantly (*p*<0.05) the least scored (5.0) compared to 035 and CICA4 (6.0) in smell. Taste was rated higher (6.0) in control 2 compared to the other two varieties (5.0), however this result was not significant (*p*>0.05). Overall liking for texture scored the lowest (5.0) in average liking of all attributes. No significant differences were found between all three varieties (*p*>0.05).

## Discussion

In some areas of Latin America and the Caribbean, the prevalence of zinc deficiency is high [4]. Intervening with biofortified rice containing enhanced zinc levels has been suggested as a cost-efficient and sustainable method of tackling this [18, 68, 69]. Thus, it could be an excellent vehicle to increase the daily zinc intake. However, a successful incorporation of biofortified crops into the target population’s diet is reliant on the consumer acceptance, which is strongly influenced by the grain quality and sensory attributes [62, 70]. The purpose of this study was to carry out a grain quality characterization and a consumer sensory acceptability study with two zinc biofortified rice varieties in Bolivia and Colombia, to identify the variety with the highest acceptance for a subsequent release to the public. The results of this study indicated that the biofortified variety T2-11 in Bolivia and the local variety control 1 were equally accepted, and that the biofortified variety 035 in Colombia had a higher overall acceptance score compared to the local consumed variety. Research shows that consumers in Colombia and Bolivia prefer ‘long and slender’ rice grains (L>7.5 mm and L/B>3), with an intermediate-high amylose content (>25%) and a low-intermediate gelatinization temperature (<74°C) [47, 51, 71]. Additionally, chalkiness is one of the key factors that determine rice quality and price, being the optimal industrial value ≤1.00 [51]. The variety MAC-18 (control 1) used in the sensory study in Bolivia harbored these characteristics (Table 1). Thus, it could be expected that a biofortified variety with a similar profile had higher chances to be accepted [20, 45, 47]. Results showed that the biofortified variety T2-11 and the control 1 presented similarities in grain size, chalkiness, amylose content and cooking time and only significantly differed in Kett degrees. The second biofortified variety tested, T2-10 presented a similar amylose content and cooking time when compared to the control, but conversely, it presented a significantly smaller size, about three times higher levels of chalkiness, and higher Kett degrees. According to the grain quality characteristic results, T2-11 seemed to have a greater chance of acceptance than T2-10. Indeed, it was the biofortified variety that scored highest in overall liking, with no significant differences compared to control 1, but significantly different compared to the other biofortified variety, T2-10 (Table 5). A significant difference (*p*<0.05) in acceptance of size between both control 1 and T2-11 compared to the other biofortified variety, T2-10, was observed (Table 6). This is not unexpected considering that T2-10 presented a smaller size.

As previously mentioned, an intermediate-high amylose content is more widely accepted in both countries, whereas a low amylose content can influence the liking score of texture attributes [48]. Our results showed that color and taste presented no significant differences between all three varieties. However, texture was significantly more accepted for the biofortified variety T2-11 and the control when compared to T2-10. This is a surprising result as the amylose content of all three rice varieties was the same. On the other hand, the biofortified variety T2-11 presented a grain size, chalkiness and cooking time very alike control 1. Thus, a similar score for both is justified. In regards to smell, the three varieties presented a similar score, but the control variety was rated significantly higher than one of the biofortified varieties. One possible explanation of this variation in rating is that rice grain characteristics of the samples may have been slightly affected by the storage time, which could have influenced the outcome of the sensory evaluation of attributes. The storage of rice for lengthy periods of time (3–6 months) prior to consumption can cause changes in smell (aroma) and color [72]. This occurs because physicochemical modifications in minor components during storage at the granule surface such lipids and proteins have an influence on grain quality properties [73]. Additionally, as a result of the chemical reaction between free amino acid groups and the carbonyl group of a reducing sugar, the Maillard reaction or non-enzymatic browning can occur. This can lead to an increase in the level of brown pigments such as melanoidins in the outer layers of the grain rice [74]. Furthermore, the profile of volatile components can also be altered with the subsequent generation of undesirable volatile lipid oxidation products [72, 75]. Although the rice varieties had been stored for a period that is on the lower end of the time spectrum for such reactions to occur -approximately 3 months at 15°C-, alterations as a result of the mentioned variables might have affected the consumer liking scores of attributes such as smell.

In Colombia, although the variety CICA4 (control 2) is one of the most sowed in the Caribbean region [76], it does not present some of the most preferred characteristics among the population. Results have shown that the rice grains were short in size, had a medium shape and a low-intermediate amylose content. The biofortified variety 035 presented the largest grain size (L/B = 2.97), which was at a level that is considered as ‘long and slender’ and therefore had high chances of being liked [47]. Control 2 on the contrary, presented the smallest size grain and scored lower in liking than the biofortified varieties, which presented a larger grain size, although with no significant differences. Surprisingly, in terms of texture, results showed that no differences were observed between all three varieties even though the amylose content of 035 and 021 was above 25% and a higher acceptance could have therefore been expected. A significant difference was found between the smell of 021, the worst rated, and the other two varieties. However, no differences were observed between this variety and the highest rated variety, 035, in any physicochemical parameter. Between control 2 and 035 no differences were observed with regards to smell.

Regarding the level of chalkiness, the biofortified varieties presented a significantly lower result when compared to the control. The variety 035 reported the lowest value with a level of 1.00, which corresponds to the optimal industrial value. A chalky appearance is associated with the development of numerous air spaces between loosely packed starch granules that can result in changes in light reflection [77]. If part of the milled rice grain is opaque rather than translucent, it is characterized as chalky and may downgrade the overall appearance of milled rice [50]. Chalkiness disappears during cooking and has no effect is therefore expected on sensory attributes [78]. Thus, although presenting the same score, the significant differences found in color between control 2 and the biofortified varieties might not have been caused by the different levels of chalkiness. Kett degrees scores were similar in all three varieties and therefore no favoritism of any variety was expected as whiteness levels were similar, which could have influenced the color outcome. Considering national consumer preferences in Colombia [47, 51], biofortified variety 035 harbored characteristics that are more favorable. The overall liking score confirmed this. In both countries all rice varieties presented similar cooking times with no significant differences. This is not unexpected since this parameter is previously studied throughout the breeding process, in order to preselect varieties with similar behaviors to the local variety.

Our results showed that the zinc concentration of the rice grain in Bolivia and Colombia was 50% higher than the local control varieties used in this study, confirming the increased levels of this micronutrient in the grain (Table 2). In Bolivia, the phytic acid levels were significantly lower in the control 1 compared to the variety T2-11, the higher scored biofortified variety in Bolivia. However, the differences in zinc and phytic acid levels between the control 1 and T2-11 did not alter the overall liking of both varieties, which presented no significant differences (Table 5). In Colombia, no significant differences were observed between the phytic acid levels of the control 2 and the higher rated biofortified variety 035, but also no significant differences in overall liking were observed. This suggest that the higher zinc levels of the variety 035 did not altered the overall liking score. An estimated PA:Zn molar ratio outcome of below 5 in all varieties in Bolivia and between 5 and 15 in all varieties in Colombia, research suggests that a moderate to high zinc absorption from zinc intake could be expected, with a minimum of 30% zinc absorption, what would be in line with EFSA guideline [56]. Thus, the biofortified rice variety with the highest score in Bolivia, T2-11, is estimated to contribute to as much as 18% and 22% of the EAR of zinc for children and women, respectively. This corresponds to an increase of a 50% in the contribution to the EAR of zinc with respect to the control variety for both women and children. In Colombia, the biofortified variety 035 may contribute up to 27% in children and 24% in women of the EAR (Table 3). Likewise, this supposes an increase of a 50% in the contribution to the EAR for both children and women with respect to the control variety. Rice is the most consumed staple in Bolivia and Colombia [15, 59], and our results indicated that 98% of participants in Bolivia and 92% of participants in Colombia reported a daily consumption of rice. The results of this study suggest that if incorporated into the daily diet, biofortified varieties may contribute towards alleviating zinc deficiencies in at risk groups such as women and children. Also, these varieties may increase the probability of meeting the EAR of their age group considering figures of zinc deficiency prevalence: 41% in the Atlantic region of Colombia where the present study was conducted and an estimate of 40% of the population with inadequate zinc intake in Bolivia [9, 15]. Additionally, it is important to mention that biofortification is a continuously improving process, with ongoing breeding for generations of biofortified varieties with increasing levels of micronutrients and nutritious qualities [18, 34].

In contrast to existing consumer literature [23, 42–44] our findings demonstrate a positive overall acceptance of the zinc biofortified rice varieties (T2-11 and 035) by consumers versus their commercial counterparts in both Bolivia and Colombia. To date, existing consumer acceptability studies on rice did not include a grain quality analysis -a major contribution to the sensory attributes of the crop such as color, size, smell, taste, and texture- in selecting biofortified varieties to be tested. This may have contributed to the contrasting results which exhibit commercial varieties (the controls) with a similar or higher overall acceptability compared to those that were nutritionally enhanced [42, 44, 79]. For example, a sensory acceptability trial in Nicaragua utilizing a rice variety (*Azucena*) with slightly increased quantities of zinc and iron found a higher acceptance of the commercial variety [42]. The same occurred in Bolivia [80]. *Azucena* is a rice typically eaten in the Philippines and has a strong aroma when cooked. However, in Nicaragua and Bolivia this type of rice is not typically consumed and therefore can result in adverse reactions in liking [42, 71]. In a sensory trial conducted in China, the control was the most liked [44]. In contrast, in Cuba, a study carried out with women showed that the nutritionally enhanced variety was more liked than the commercial. However, it should be noted that the commercial variety utilized in this case was an imported variety, thus was most likely not consumed regularly [43].

There is substantial evidence proving the efficacy and effectiveness of biofortified crops in increasing micronutrient intake and improving health status in humans [81–89]. Hitherto, only two effectiveness studies have been conducted with biofortified crops, both assessing provitamin A biofortified sweet potato. The conclusion was that the consumption of this crop on a larger scale increased vitamin A intake in children and women [90, 91]. Although efficacy studies on zinc biofortified crops are limited, in part due to the lack of sensitive biomarkers to measure increases in zinc status from dietary zinc, results in existing literature have shown that zinc intake can be increased via regular consumption of zinc biofortified rice [40, 92]. To date, there is only one existing efficacy study that has evaluated zinc biofortified crops, which was carried out in Delhi, India. In this study, after 6 months of zinc biofortified wheat flour consumption, a decline in morbidity of children and non-pregnant non-lactating women was observed alongside a reduction in days with pneumonia and vomiting in children, and less days with fever reported in women [93]. Although the number of studies conducted with zinc crops are sparse, evidence has shown that micronutrient levels have increased after consuming biofortified crops for an extended period [94]. Additionally, research is underway to identify novel zinc biomarkers, with promising advances [95–97]. Rice crops biofortified with zinc may have the potential to achieve similar results and efficacy studies are therefore desirable in countries such as Bolivia or Colombia.

Finally, research has shown that there is a higher acceptance rate when nutritional information is provided along-side recommendations of introducing biofortified foods into the daily diet [98, 99]. Thus, the combined effort of introducing biofortified crops together with the promotion of nutritionally diversified diets is encouraged [100, 101].

### Limitations and strengths

This study determined the acceptability of rice varieties to select one for release in Bolivia and Colombia. However, as the trials in both countries were carried out in the selected departments where the rice varieties were planned for release, results may not be generalizable to the whole country.

Crops are subjected to genotype by environment interaction (GxE) and to agronomic management practices. Environmental factors such as soil redox potential, pH, soil structure and texture, organic matter content and climate conditions, among others, influence GxE interaction and may affect rice zinc levels accumulation [102, 103]. This is a consequence of the alteration of quantitative trait locus (QTL) responsible for the absorption and translocation of zinc to the grain in biofortified varieties [68]. Thus, slight variations in zinc levels can occur when the rice varieties are grown in departments other than the ones where the study was conducted, or if agronomic management practices are different to the recommended are followed.

The average age of the participants in this study could be viewed as a limitation. Participant mean age recorded was young to middle age adults; 34 years old in Bolivia and 24 years old in Colombia, which suggests that results cannot be extrapolated to all age groups in the study regions. However, women of reproductive age, defined by WHO as 15–49 years old, are one of the highest groups at risk of zinc deficiencies [104]. Additionally, there may be a risk of selection bias, as the average participant in this study reported progression to further education. This, however, does not affect the benefit that biofortified varieties would have in the regions it will be released. Nevertheless, the acceptance results in this case are not generalizable to the whole population.

Another limitation that was encountered that there is no guideline available to estimate the zinc absorption from a single crop. However, to estimate the absorption of zinc from a specific diet there are multiple sources available [105]. Thus, for this study, a 30% absorption was assumed according to EFSA, who provide the most recently reviewed calculations [56]. However, as this is based on a mixed diet and the calculations in this study were focused on a single crop this percentage may vary slightly in a whole mixed diet.

A final limitation was found in our capacity to estimate the contribution to the EAR given that there is no official data for rice consumption neither in children nor in women in Bolivia. To calculate it, data available for children in six South American countries (Argentina, Brazil, Colombia, Chile, Peru, and Uruguay) was utilized [57]. For women, the consumption per capita data extracted from the FAO food balance sheets was used [28].

## Conclusions

The overall acceptability of the biofortified variety T2-11 and control variety for Bolivia was similar, highlighting that no overall difference in sensory attributes or grain quality was detected. For Colombia, the biofortified variety 035 scored the highest, suggesting that it was more accepted than the control variety. Our results indicate that the biofortified varieties have a strong potential to be accepted by consumers following release of the selected varieties. As can be reported in this study, grain quality of rice is of high importance for consumers, thus it is recommended that grain quality should be a focal point in crop breeding stages of biofortified rice varieties. The sensory acceptance of the zinc biofortified rice varieties by consumers endorses their release in Bolivia and Colombia, with potential to reduce zinc deficiencies when incorporated into the daily diet.

There is a prospective for a higher impact if policy makers and public leaders encourage the implementation of biofortified crops into the food system value chains and nutrition agendas. Moreover, research shows that nutritional education programs which aim at informing the health and nutritional benefits of biofortified crops have improved acceptability and enhanced the likelihood of mothers integrating it into child diets. Thus, it is recommended that following the release of biofortified varieties in Bolivia and Colombia, information sessions and educational programs are provided.

## Supporting information

S1 File Questionnaire for the sensory evaluation of biofortified rice (Spanish and English). (PDF)

## References

[1] Gödecke T, Stein AJ, Qaim M. The global burden of chronic and hidden hunger: Trends and determinants. Global Food Secur. 2018; 17, 21–29. 10.1016/j.gfs.2018.03.004

[2] Rehman A, Farooq M, Ozturk L, Asif M, Siddique KH. Zinc nutrition in wheat-based cropping systems. Plant Soil. 2018; 422(1–2), 283–315. 10.1007/s11104-017-3507-3

[3] Bouis HE, Islam Y. Biofortification: Leveraging Agriculture to Reduce Hidden Hunger. In: Shenggen F, Pandya-Lorch R, editors. Reshaping agriculture for nutrition and health. Washington (DC): International Food Policy Research Institute (IFPRI); 2012 Chapter 10, pp. 83–92.

[4] Bailey RL, West KP Jr, Black RE. The epidemiology of global micronutrient deficiencies. Ann. Nutr. Metab. 2015; 66(Suppl. 2), 22–33. 10.1159/000371618 26045325

[5] Zaman QU, Aslam Z, Yaseen M, Ihsan MZ, Khaliq A, Fahad S, et al Zinc biofortification in rice: leveraging agriculture to moderate hidden hunger in developing countries. Arch. Agron. Soil Sci. 2018; 64(2), 147–161. 10.1080/03650340.2017.1338343

[6] Ackland ML, Michalczyk AA. Zinc and infant nutrition. Arch. Biochem. Biophys. 2016; 611, 51–57. 10.1016/j.abb.2016.06.011 27317042

[7] Phattarakul N, Rerkasem B, Li LJ, Wu LH, Zou CQ, Ram H, et al Biofortification of rice grain with zinc through zinc fertilization in different countries. Plant Soil. 2012; 361(1–2), 131–141. 10.1007/s11104-012-1211-x

[8] Yang R, Sun Y, Gu Z. Zinc Accumulation and Distribution in Germinated Brown Rice. Food Sci. Technol. Res. 2018; 24(3), 369–376. 10.3136/fstr.24.369

[9] Cediel G, Olivares M, Brito A, Cori H, López de Romaña D. Zinc deficiency in Latin America and the Caribbean. Food Nutr. Bull. 2015; 36(2_suppl), S129-S138. 10.1177/0379572115585781 26125198

[10] Ruz M, Solomons NW. A Vision for Nutritional Research for the Latin American Region. Food Nutr. Bull. 2019; 40(1), 14–25. 10.1177/0379572119832780 30827120

[11] Gupta S, Brazier AKM, Lowe NM. Zinc deficiency in low- and middle-income countries: prevalence and approaches for mitigation. J Hum Nutr Diet. 2020; 33(5), 624–643. 10.1111/jhn.12791 32627912

[12] López de Romaña D, Cediel G. Current Situation of Micronutrients in Latin America and the Caribbean. In: Irizarry L, Prost MA, Murillo D, editors. Scaling Up Rice Fortification in Latin America and the Caribbean. Panama: World Food Programme (WFP); 2017 pp. 122–135.

[13] Lazarte C. Nutritional Assessment in a Rural Area of Bolivia. A Study of Zinc and Iron Deficiencies and Bioavailability. Ph.D. Thesis, Lund University. 2014. Available from: https://lup.lub.lu.se/search/ws/files/5436855/4195866.pdf

[14] Grandy G, Weisstaub G, López de Romana D. Deficiencia de hierro y zinc en niños. Rev. Bol. Ped. 2010; 49(1), 25–31.

[15] Ministerio de Salud y Protección Social, República de Colombia (MINSALUD); Departamento Administrativo para la Prosperidad Social; Instituto Colombiano de Bienestar Familiar (ICBF); Instituto Nacional de Salud (INS); Universidad Nacional de Colombia. Encuesta Nacional de la Situación Nutricional en Colombia–ENSIN 2015. Bogotá: Ministerio de Salud y Protección Social; 2019.

[16] Pinzón-Rondón AM, Hoyos-Martínez A, Parra-Correa D, Pedraza-Flechas AM, Ruiz-Sternberg AM. Association of nutritional support programs with zinc deficiency in Colombian children: a cross-sectional study. BMC Nutr. 2019; 5(1), 42 10.1186/s40795-019-0305-8 32153955 PMC7050802

[17] Lazarte CE, Soto A, Alvarez L, Bergenståhl B, Medrano N, Granfeldt Y. Nutritional status of children with intestinal parasites from a tropical area of Bolivia, emphasis on zinc and iron status. Food Nutr. Sci. 2015; 6(04), 399 10.4236/fns.2015.64041

[18] Bouis HE, Hotz C, McClafferty B, Meenakshi JV, Pfeiffer WH. Biofortification: a new tool to reduce micronutrient malnutrition. Food Nutr. Bull. 2011; 32(1_suppl1), S31–S40. 10.1177/15648265110321S105 21717916

[19] Singh SS, Hazra KK, Praharaj CS, Singh U. Biofortification; pathway ahead and future challenges. In: Singh U, Praharaj CS, Singh SS, Singh NP, editors. Biofortification of Food Crops. New Delhi: Springer; 2016 pp. 479–492.

[20] Saltzman A, Birol E, Bouis HE, Boy E, de Moura FF, Islam Y, et al Biofortification: progress toward a more nourishing future. Global Food Secur. 2013; 2(1), 9–17.

[21] Noulas C, Tziouvalekas M, Karyotis T. Zinc in soils, water and food crops. J. Trace Elem. Med. Biol. 2018; 49, 252–260. 10.1016/j.jtemb.2018.02.009 29472130

[22] Garg M, Sharma N, Sharma S, Kapoor P, Kumar A, Chunduri V, et al Biofortified Crops Generated by Breeding, Agronomy, and Transgenic Approaches Are Improving Lives of Millions of People around the World. Front. Nutr. 2018; 5(12), 1–33. 10.3389/fnut.2018.00012 29492405 PMC5817065

[23] Talsma EF, Melse-Boonstra A, Brouwer ID. Acceptance and adoption of biofortified crops in low-and middle-income countries: a systematic review. Nutr. Rev. 2017; 75(10), 798–829. 10.1093/nutrit/nux037 29028269 PMC5914320

[24] Heck S, Campos H, Barker I, Okello JJ, Bran A, Boy E, et al Resilient agri-food systems for nutrition amidst COVID-19: evidence and lessons from food-based approaches to overcome micronutrient deficiency and rebuild livelihoods after crises. Food Sec. 2020; 12, 823–830. 10.1007/s12571-020-01067-2 32839664 PMC7381414

[25] Jha AB, Warkentin TD. Biofortification of Pulse Crops: Status and Future Perspectives. Plants. 2020; 9(1), 73 10.3390/plants9010073 31935879 PMC7020478

[26] Meenakshi JV, Johnson NL, Victor MM, De Groote H, Josyline J, Yanggen DR, et al How Cost-Effective is Biofortification in Combating Micronutrient Malnutrition? An Ex ante Assessment. World Dev. 2010; 38(1), 64–75. 10.1016/j.worlddev.2009.03.014

[27] Liu K, Zheng J, Chen F. Effect of domestic cooking on rice protein digestibility. Food Sci. Nutr. 2019; 7(2), 608–616. 10.1002/fsn3.884 30847140 PMC6392838

[28] FAO, FAOSTAT—Food and Agriculture Data. [cited 2020 Mar 5]. Available from: http://www.fao.org/faostat/en/#home

[29] Andersson MS, Saltzman A, Virk PS, Pfeiffer WH. Progress update: crop development of biofortified staple food crops under HarvestPlus. Afr. J. Food Agric. Nutr. Dev. 2017; 17(2), 11905–11935.

[30] Liang J, Li Z, Tsuji K, Nakano K, Nout MJR, Hamer RJ. Milling characteristics and distribution of phytic acid and zinc in long-, medium- and short-grain rice. J. Cereal Sci. 2008; 48(1), 83–91. 10.1016/j.jcs.2007.08.003

[31] Talsma EF, Gallego-Castillo S, Carrillo P, Londoño LF, Grenier C, Palacios-Rojas N. Retention of zinc in biofortified rice and maize during processing and cooking. Proceedings of the 4th Micronutrient Forum Global Conference; 2016 Oct 23–28; Cancun, MX. Abstract No. 0290.

[32] Haddad L, Hawkes C, Waage J, Webb P, Godfray C, Toulmin C. Food systems and diets: Facing the challenges of the 21st century. London: Global Panel on Agriculture and Food Systems for Nutrition; 2016.

[33] Kennedy G, Moursi M. Dietary diversity and biofortification: Closer than you think. HarvestPlus Policy Brief. Washington (DC): International Food Policy Research Institute (IFPRI); 2015.

[34] Herrington C, Lividini K, Angel MD, Birol E. Prioritizing countries for biofortification interventions: Biofortification Priority Index Second Edition (BPI 2.0). HarvestPlus Working Paper No. 40. Washington (DC): International Food Policy Research Institute (IFPRI); 2019.

[35] Whiting SJ, Berhanu G, Haileslassie HA, Henry CJ. Pulses and Mineral Bioavailability in Low Income Countries. In: Dahl W, editor. Health Benefits of Pulses. Basel, Switzerland: Springer; 2019 pp. 43–53.

[36] Azman Halimi R, Barkla BJ, Andrés‐Hernandéz L, Mayes S, King GJ. Bridging the food security gap: an information‐led approach to connect dietary nutrition, food composition and crop production. J. Sci. Food Agric. 2020; 100(4), 1495–1504. 10.1002/jsfa.10157 31756768

[37] Yadav N, Kaur D, Malaviya R, Saini P, Anjum S. Enhancement in mineral bioavailability of extruded pulses with reduced antinutrients. Br. Food J. 2019; 121(11), 2967–2978. 10.1108/BFJ-04-2019-0236

[38] Gibson RS, Raboy V, King JC. Implications of phytate in plant-based foods for iron and zinc bioavailability, setting dietary requirements, and formulating programs and policies. Nutr. Rev. 2018; 76(11), 793–804. 10.1093/nutrit/nuy028 30010865

[39] Liu D, Liu Y, Zhang W, Chen X, Zou C. Agronomic approach of zinc biofortification can increase zinc bioavailability in wheat flour and thereby reduce zinc deficiency in humans. Nutrients. 2017; 9(5), 465 10.3390/nu9050465 28481273 PMC5452195

[40] Bouis HE, Saltzman A. Improving nutrition through biofortification: A review of evidence from HarvestPlus, 2003 through 2016. Glob. Food Sec. 2017; 12, 49–58. 10.1016/j.gfs.2017.01.009 28580239 PMC5439484

[41] King JC, Vorster HH, Tome DG. Nutrient intake values (NIVs): a recommended terminology and framework for the derivation of values. Food Nutr. Bull. 2007; 28(1_suppl1), S16–S26. 10.1177/15648265070281S103 17521116

[42] Montecinos KLG, Godoy JAG, Centeno PMC, Pachón H. Sensory evaluation of rice (*Oryza sativa*) variety Azucena in the Autonomous Region of the North Atlantic in Nicaragua. Perspect. Hum. Nutr. 2011; 13(2), 135–146.

[43] Padrón VP, Crestelo ES, Caraballo RA, Pachón H, Martínez CP. Preference and acceptability of the IACuba 30 rice variety with high iron and zinc content by pregnant women in Cuba. Perspect. Nut. Hum. 2011; 13, 123–134.

[44] De Bree A. Acceptability of Zinc Fortified Rice by Women in Rural Chuzhou District, China. M.Sc. Thesis, Wageningen University. 2009.

[45] Champagne ET, Bett-Garber KL, Fitzgerald MA, Grimm CC, Lea J, Ohtsubo K, et al Important sensory properties differentiating premium rice varieties. Rice. 2010; 3, 270–281. 10.1007/s12284-010-9057-4

[46] Fitzgerald M. Rice: Grain-quality characteristics and management of quality requirements. In: Wrigley C, Batey I, Miskelly D, editors. Cereal Grains. 2nd ed. Cambridge: Woodhead Publishing; 2017 pp. 291–315.

[47] Calingacion M, Laborte A, Nelson A, Resurreccion A, Concepcion JC, Daygon VD, et al Diversity of global rice markets and the science required for consumer-targeted rice breeding. PLOS ONE. 2014; 9(1), e85106 10.1371/journal.pone.0085106 24454799 PMC3893639

[48] Dela Cruz ND, Khush GS. Rice grain quality evaluation procedures. In: Singh RK, Singh US, Khush GS, editors. Aromatic rices. New Delhi: Oxford & IBH Publishing Co.; 2000 pp. 15–28.

[49] De Groote H, Gunaratna NS, Okuro JO, Wondimu A, Chege CK, Tomlins K. Consumer acceptance of quality protein maize (QPM) in East Africa. J. Sci. Food Agric. 2014; 94(15), 3201–3212. 10.1002/jsfa.6672 24659349

[50] Bergman C. Rice end-use quality analysis. In: Bao J, editor. Rice Chemistry and Technology. 4th ed. Duxford: Woodhead Publishing; 2019 pp. 273–337.

[51] Martínez CP, Cuevas FE, Medina LM. Evaluación de la calidad culinaria y molinera del arroz. Guía de estudio para ser usada como complemento de la unidad audio tutorial sobre el mismo tema. Cali: Centro Internacional de Agricultura Tropical (CIAT); 1989.

[52] Poritosh R, Daisuke N, Takahiro O, Hiroshi O, Manasikan T, Nobutaka N, et al Cooking properties of different forms of rice cooked with an automatic induction heating system rice cooker. As. J. Food Ag-Ind. 2010; 3(04), 373–388.

[53] Paltridge NG, Palmer LJ, Milham PJ, Guild GE, Stangoulis JC. Energy-dispersive X-ray fluorescence analysis of zinc and iron concentration in rice and pearl millet grain. Plant Soil. 2012; 361(1–2), 251–260. 10.1007/s11104-011-1104-4

[54] Latta M, Eskin M. A simple and rapid colorimetric method for phytate determination. J. Agric. Food Chem. 1980; 28(6), 1313–1315. 10.1021/jf60232a049

[55] Coelho CMM, Bellato CM, Garcia AKM, Vitorello VA, Azevedo RA. Variation in phytate accumulation in common bean (*Phaseolus vulgaris* L.) fruit explants. Braz. Arch. Biol. Technol. 2008; 51(1), 163–173. 10.1590/S1516-89132008000100020

[56] European Food Safety Authority. Scientific opinion on Dietary Reference values for zinc. EFSA Panel on Dietetic Products, Nutrition and Allergies (NDA). EFSA J. 2014; 12, 1–76. 10.2903/j.efsa.2014.3660 PMC13136992 42087959

[57] Saravia L, González‐Zapata LI, Rendo‐Urteaga T, Ramos J, Collese TS, Bove I, et al Development of a food frequency questionnaire for assessing dietary intake in children and adolescents in South America. Obesity. 2018; 26, S31–S40. 10.1002/oby.22114 29464920

[58] Zapata-Caldas E, Hyman G, Pachón H, Monserrate FA, Varela LV. Identifying candidate sites for crop biofortification in Latin America: case studies in Colombia, Nicaragua and Bolivia. Int. J. Health Geographics. 2009; 8(1), 29 10.1186/1476-072X-8-29 19454034 PMC2689862

[59] CGIAR, Ricepedia.org. [cited 2019 Sep 18]. Available from: http://ricepedia.org/bolivia

[60] Lawless HT, Heymann H. Sensory evaluation of food: principles and practices. 2nd ed. Food Science Text Series New York: Springer; 2010 pp. 325–344.

[61] Ares G, Tárrega A, Izquierdo L, Jaeger SR. Investigation of the number of consumers necessary to obtain stable sample and descriptor configurations from check-all-that-apply (CATA) questions. Food Qual. Prefer. 2014; 31, 135–141. 10.1016/j.foodqual.2013.08.012

[62] Beintema JJ, Gallego‐Castillo S, Londoño‐Hernandez LF, Restrepo‐Manjarres J, Talsma EF. Scaling‐up biofortified beans high in iron and zinc through the school‐feeding program: A sensory acceptance study with schoolchildren from two departments in southwest Colombia. Food Sci. Nutr. 2018; 6(4), 1138–1145. 10.1002/fsn3.632 29983978 PMC6021705

[63] Delarue J. The use of rapid sensory methods in R&D and research: an introduction. In: Delarue J, Lawlor B, Rogeaux M, editors. Rapid sensory profiling techniques and related Methods. Applications in new product development and consumer research. Cambridge: Woodhead Publishing; 2014 pp. 3–22.

[64] Mestres C, Brifazz A, Valentin D. Rice cooking and sensory quality. In: Bao J, editor. Rice Chemistry and Technology. 4th ed. Duxford, UK: Woodhead Publishing; 2019 pp. 400–417.

[65] De Kock HL, Kamdem Mademgne JD. Designing Consumer Research Studies for Low-Income Populations. In: Ares G, Varela P, editors. Methods in Consumer Research, Volume 2: Alternative Approaches and Special Applications Cambridge: Woodhead Publishing; 2018 Chapter 15, pp. 373–391.

[66] Carrillo P, Gallego S, Talsma EF. Sensory tests in biofortified crops. Field manual. Palmira: HarvestPlus; 2016.

[67] Norhaizan ME, Nor Faizadatul Ain AW. Determination of phytate, iron, zinc, calcium contents and their molar ratios in commonly consumed raw and prepared food in Malaysia. Mal. J. Nutr. 2009; 15(2), 213–222. 22691819

[68] Swamy BPM, Descalsota GIL, Nha CT, Amparado A, Inabangan-Asilo MA, Manito C, et al Identification of genomic regions associated with agronomic and biofortification traits in DH populations of rice. PLoS ONE. 2018; 13(8): e0201756 10.1371/journal.pone.0201756 30096168 PMC6086416

[69] Majumder S, Datta K, Datta SK. Rice Biofortification: High Iron, Zinc, and Vitamin-A to Fight against “Hidden Hunger”. Agronomy. 2019; 9(803), 1–22. 10.3390/agronomy9120803

[70] Stevens R, Winter-Nelson A. Consumer acceptance of provitamin A-biofortified maize in Maputo, Mozambique. Food Policy. 2008; 33(4), 341–351. 10.1016/j.foodpol.2007.12.003

[71] Viruez J, Taboada R. Producción de arroz en Bolivia: Conocimiento técnico para un manejo eficiente y rentable. 1st ed. Santa Cruz, Bolivia: Programa Arroz CIAT-Santa Cruz; 2013 pp. 40–41.

[72] Tananuwong K, Lertsiri S. Changes in volatile aroma compounds of organic fragrant rice during storage under different conditions. J. Sci. Food Agric. 2010; 90(10), 1590–1596. 10.1002/jsfa.3976 20564458

[73] Kongkiattikajorn J. Effect of storage time and temperature on volatile aroma compounds and physicochemical properties of rice. Kasetsart J. (Nat. Sci.). 2008; 42, 111–117.

[74] Perez-Locas C, Yaylayan V. The Maillard reaction and food quality deterioration. In: Risbo J, Skibsted LH, Andersen ML, editors. Chemical Deterioration and Physical Instability of Food and Beverages: Cambridge: Woodhead Publishing; 2010 pp. 70–94.

[75] Tong C, Bao J. Rice lipids and rice bran oil. In: Bao J, editor. Rice Chemistry and Technology. 4th ed. Duxford: Woodhead Publishing; 2019 pp. 144–152.

[76] Aramendiz Tatis H, Espitia Camacho M, Cardona Ayala C. Adaptation of irrigated rice (*Oryza sativa* L.) in the Colombian Caribbean. Agronomic Act. 2011; 60 (1), 01–12.

[77] Yoshioka Y, Iwata H, Tabata M, Ninomiya S, Ohsawa R. Chalkiness in rice: Potential for evaluation with image analysis. Crop Sci. 2007; 47, 2113–2120.

[78] Santos MV, Cuevas RPO, Sreenivasulu N, Molina L. Measurement of rice grain dimensions and chalkiness, and rice grain elongation using image analysis. In: Sreenivasulu N, editor. Rice Grain Quality: Methods in Molecular Biology New York: Humana Press; 2019 Volume 1892, pp. 99–108. 10.1007/978-1-4939-8914-0_6 30397802

[79] Vergara O, Buitrago IC, Henriquez T, de Caballero EV, de Torres EM, Espinosa J, et al Evaluación sensorial de arroz biofortificado, variedad IDIAP Santa Cruz 11, en granjas autosostenibles del Patronato de Nutrición en la Provincia de Coclé, Panamá. Perspect. Nutr. Hum. 2011; 13, 147–160. 10.17533/udea.penh

[80] Taboada R, Viruez J, Martínez C, Borrero J. Informe técnico proyecto AgroSalud en el cultivo de arroz. CIAT Santa Cruz–ASPAR. Santa Cruz, Bolivia: Programa Arroz CIAT-Santa Cruz; 2010.

[81] Finkelstein JL, Mehta S, Udipi SA, Ghugre PS, Luna SV, Wenger MJ, et al A Randomized Trial of Iron-Biofortified Pearl Millet in School Children in India. J. Nutr. 2015; 145(7), 1576–1581. 10.3945/jn.114.208009 25948782

[82] Haas JD, Luna SV, Lung'aho MG, Wenger MJ, Murray-Kolb LE, Beebe S, et al Consuming Iron Biofortified Beans Increases Iron Status in Rwandan Women after 128 Days in a Randomized Controlled Feeding Trial. J. Nutr. 2016; 146(8), 1586–1592. 10.3945/jn.115.224741 27358417

[83] Talsma EF, Brouwer ID, Verhoef H, Mbera GNK, Mwangi AM, Demir AY, et al Biofortified yellow cassava and vitamin A status of Kenyan children: a randomized controlled trial. Am. J. Clin. Nutr. 2016; 103(1), 258–267. 10.3945/ajcn.114.100164 26675768

[84] Palmer AC, Healy K, Barffour MA, Siamusantu W, Chileshe J, Schulze KJ, et al Provitamin A Carotenoid–Biofortified Maize Consumption Increases Pupillary Responsiveness among Zambian Children in a Randomized Controlled Trial. J. Nutr. 2016; 146(12), 2551–2558. 10.3945/jn.116.239202 27798345

[85] Murray-Kolb LE, Wenger MJ, Scott SP, Rhoten SE, Lung'aho MG, Haas JD. Consumption of Iron-Biofortified Beans Positively Affects Cognitive Performance in 18- to 27-Year-Old Rwandan Female College Students in an 18-Week Randomized Controlled Efficacy Trial. J. Nutr. 2017; 147(11), 2109–2117. 10.3945/jn.117.255356 28954841 PMC5657139

[86] Scott SP, Murray-Kolb LE, Wenger MJ, Udipi SA, Ghugre PS, Boy E, et al Cognitive Performance in Indian School-Going Adolescents Is Positively Affected by Consumption of Iron-Biofortified Pearl Millet: A 6-Month Randomized Controlled Efficacy Trial. J. Nutr. 2018; 148(9), 1462–1471. 10.1093/jn/nxy113 30016516

[87] Govender L, Pillay K, Siwela M, Modi AT, Mabhaudhi T. Improving the Dietary Vitamin A Content of Rural Communities in South Africa by Replacing Non-Biofortified White Maize and Sweet Potato with Biofortified Maize and Sweet Potato in Traditional Dishes. Nutrients. 2019a; 11(6), 1198 10.3390/nu11061198 31141908 PMC6628247

[88] Wenger MJ, Rhoten SE, Murray-Kolb LE, Scott SP, Boy E, Gahutu JB, et al Changes in Iron Status Are Related to Changes in Brain Activity and Behavior in Rwandan Female University Students: Results from a Randomized Controlled Efficacy Trial Involving Iron-Biofortified Beans. J Nutr. 2019; 149(4), 687–697. 10.1093/jn/nxy265 30926992 PMC6461719

[89] Luna SV, Pompano LM, Lung´aho M, Gahutu JB, Haas JD. Increased Iron Status during a Feeding Trial of Iron-Biofortified Beans Increases Physical Work Efficiency in Rwandan Women. J Nutr. 2020; 150(5), 1093–1099. 10.1093/jn/nxaa016 32006009 PMC7198300

[90] Hotz C, Loechl C, De Brauw A, Eozenou P, Gilligan D, Moursi M, et al A large-scale intervention to introduce orange sweet potato in rural Mozambique increases vitamin A intakes among children and women. Br. J. Nutr. 2012a; 108(1), 163–176. 10.1017/S0007114511005174 22018075

[91] Hotz C, Loechl C, Lubowa A, Tumwine JK, Ndeezi G, Masawi AN, et al Introduction of β-carotene–rich orange sweet potato in rural Uganda resulted in increased vitamin A intakes among children and women and improved vitamin A status among children. J. Nutr. 2012b; 142(10), 1871–1880. 10.3945/jn.111.151829 22875553

[92] Brnić M, Wegmüller R, Melse-Boonstra A, Stomph T, Zeder C, Tay FM, et al Zinc absorption by adults is similar from intrinsically labeled zinc-biofortified rice and from rice fortified with labeled zinc sulfate. J. Nutr. 2016; 146(1), 76–80. 10.3945/jn.115.213421 26674764

[93] Sazawal S, Dhingra U, Dhingra P, Dutta A, Deb S, Kumar J, et al A. Efficacy of high zinc biofortified wheat in improvement of micronutrient status, and prevention of morbidity among preschool children and women-a double masked, randomized, controlled trial. Nutr. J. 2018; 17(1), 86 10.1186/s12937-018-0391-5 PMC6139156 30219062

[94] Boy E, Haas JD, Petry N, Cercamondi CI, Gahutu JB, Mehta S, et al Efficacy of iron-biofortified crops. Afr. J. Food Agric. Nutr. Dev. 2017; 17(2), 11879–11892.

[95] King JC, Brown KH, Gibson RS, Krebs NF, Lowe NM, Siekmann JH, et al Biomarkers of Nutrition for Development (BOND)—Zinc Review. J Nutr. 2016; 146(4), 858S–885S. 10.3945/jn.115.220079 PMC4807640 26962190

[96] Knez M, Stangoulis JCR, Glibetic M, Tako E. The Linoleic Acid: Dihomo-γ-Linolenic Acid Ratio (LA:DGLA)—An Emerging Biomarker of Zn Status. Nutrients. 2017; 9(8), 825 10.3390/nu9080825 28763004 PMC5579618

[97] Reis BZ, Vieira DADS, Maynard DDC, Silva DGD, Mendes-Netto RS, Cozzolino SMF. Zinc nutritional status influences ZnT1 and ZIP4 gene expression in children with a high risk of zinc deficiency. J Trace Elem Med Biol. 2020; 61:126537 10.1016/j.jtemb.2020.126537 32388102

[98] Govender L, Pillay K, Siwela M, Modi AT, Mabhaudhi T. Consumer Perceptions and Acceptability of Traditional Dishes Prepared with Provitamin A-Biofortified Maize and Sweet Potato. Nutrients. 2019b; 11(7), 1577 10.3390/nu11071577 31336921 PMC6682973

[99] Ansari SA, Thapa S. Biofortification of Food Crops: An Approach towards Improving Nutritional Security in South Asia. IJAAST. 2019; 6(12), 23–33.

[100] Sharma P, Aggarwal P, Kaur A. Biofortification: A new approach to eradicate hidden hunger. Food Rev. Int. 2017; 33(1), 1–21. 10.1080/87559129.2015.1137309

[101] Johns T, Eyzaguirre PB. Biofortification, biodiversity and diet: a search for complementary applications against poverty and malnutrition. Food Policy. 2007; 32(1), 1–24. 10.1016/j.foodpol.2006.03.014

[102] Inabangan-Asilo MA, Swamy BPM, Amparado AF, Descalsota-Empleo GIL, Arocena EC, Reinke R. Stability and G x E analysis of zinc-biofortified rice genotypes evaluated in diverse environments. Euphytica. 2019; 215(61), 1–17. 10.1007/s10681-019-2384-7

[103] Calayugan MIC, Formantes AK, Amparado A, Descalsota-Empleo GI, Nha CT, Inabangan-Asilo MA, et al Genetic Analysis of Agronomic Traits and Grain Iron and Zinc Concentrations in a Doubled Haploid Population of Rice (Oryza sativa L.). Sci. Rep. 2020; 10(2283), 1–14. 10.1038/s41598-020-59184-z 32042046 PMC7010768

[104] Cetin I, Bühling K, Demir C, Kortam A, Prescott SL, Yamashiro Y, et al Impact of micronutrient status during pregnancy on early nutrition programming. Ann. Nutr. Metab. 2019; 74, 269–278. 10.1159/000499698 30939482

[105] Gibson RS, King JC, Lowe N. A Review of Dietary Zinc Recommendations. Food Nutr. Bull. 2016; 37(4) 443–460. 10.1177/0379572116652252 27312357

