# Supplementary material for: The acceptance of zinc biofortified rice in Latin America: A consumer sensory study and grain quality characterization
Source: PLoS One. 2020 Nov 11;15(11):e0242202. doi: 10.1371/journal.pone.0242202 (PMC7657500; doi:10.1371/journal.pone.0242202)
Supplement: S1 File — (PDF) [file pone.0242202.s001.pdf]

ENCUESTA PARA LA EVALUACIÓN SENSORIAL DE ARROZ BIOFORTIFICADO

Fecha: _______________ Horario: ______________ Código del participante: _______________

DATOS GENERALES

1. Sexo: a) Hombre ____ b) Mujer ____

2. Edad: _______ años

3. Estado civil: a) Soltero/a ____ b) Casado/a o unión libre ____

4. Educación: a) Ninguna ____ b) Primaria ____ c) Secundaria ____ d) Superior ____

5. Ocupación principal:
a) Empleado ____ b) Ama de casa ____ c) Agricultor ____ d) Ninguna ____

6. ¿Ha fumado en la última hora? a) Si ____ b) No ____

7. Por favor seleccione los alimentos/bebidas que haya consumido en la última hora?
a) Café/Té negro ____ b) Bebidas alcohólicas ____ c) Bebidas gaseosas/Jugo ____
d) Dulces/Mentas/Chicles ____ d) Otras comidas ____

8. ¿Con qué frecuencia consume arroz?
a) Todos los días ____ b) 1-2 veces por semana ____ c) 1-2 veces por mes ____
d) Nunca o casi nunca ____

9. ¿Cuáles aspectos tiene en cuenta al momento de comprar el arroz que usted consume?
a) Precio ____ b) Marca ____ c) Diseño del empaque ____ d) Contenido neto ____
e) Tipo de arroz (Por ej. *Integral*, *Parbolizado*) ____

PRUEBA SENSORIAL

Recuerde enjuagar su boca antes de empezar y tomar agua entre cada muestra.
Si tiene alguna pregunta, no dude en hacerla.

Frente a usted tiene tres (3) muestras de ARROZ, por favor pruébelas de izquierda a derecha o en el orden establecido. Señale con una (X) la carita que más represente lo que le pareció cada una.

POR FAVOR RESPONDA SOLO UNA OPCIÓN

	Me disguta mucho	Me disgusta	Me disgusta poco	Ni me gusta Ni me disgusta	Me gusta poco	Me gusta	Me gusta mucho
**Color**							
**Tamaño**							
**Olor**							
**Sabor**							
**Textura**							

	Me disguta mucho	Me disgusta	Me disgusta poco	Ni me gusta Ni me disgusta	Me gusta poco	Me gusta	Me gusta mucho
**Color**							
**Tamaño**							
**Olor**							
**Sabor**							
**Textura**							

	Me disguta mucho	Me disgusta	Me disgusta poco	Ni me gusta Ni me disgusta	Me gusta poco	Me gusta	Me gusta mucho
**Color**							
**Tamaño**							
**Olor**							
**Sabor**							
**Textura**							

¿Tiene algún comentario? Por favor escriba aquí:

¡MUCHAS GRACIAS POR SU PARTICIPACIÓN!

QUESTIONNAIRE FOR THE SENSORY EVALUATION OF BIOFORTIFIED RICE

Date: ________________ Time: _______________ Participant code: ________________

GENERAL INFORMATION

1. Sex: a) Man ____ b) Woman ____

2. Age: ________ years old

3. Marital status: a) Single ____ b) Married or in a union ____

4. Education: a) None ____ b) Primary School ____ c) Secondary School ____

d) Higher Education____

5. Main occupation:

a) Employed ____ b) Housewife ____ c) Farmer ____ d) None____

6. Have you smoked in the last hour? a) Yes ____ b) No ____

7. Please select the food/drinks you have consumed during the last hour

a) Coffee/Black tea ____ b) Alcoholic drinks ____ c) Soft drinks/Juice ____

d) Sweets/Candys/Chewing gum ____ d) Other food ____

8. How frequently do you consume rice?

a) Everyday ____ b) 1-2 times per week ____ c) 1-2 times per month ____

d) Never or almost never ____

9. Which aspects do you consider to be important to you when you buy rice?

a) Price ____ b) Brand ____ c) Packaging design ____ d) Net content ____

e) Rice type (e.g. Brown, Parboiled) ____

SENSORY TEST

Remember to rinse your mouth with water before starting the test and in between each sample.
If you have any inquiry, please do not hesitate in contacting us.
In front of you, you have three (3) RICE simples, please taste them from the left to the right or in the established order. Please mark with an (X) the face that best represents your opinion of each attribute.

PLEASE ANSWER ONLY ONE OPTION

	Dislike very much	Dislike moderately	Dislike slightly	Neither like nor dislike	Like slightly	Like moderately	Like very much
**Color**							
**Size**							
**Smell**							
**Taste**							
**Texture**							

	Dislike very much	Dislike moderately	Dislike slightly	Neither like nor dislike	Like slightly	Like moderately	Like very much
**Color**							
**Size**							
**Smell**							
**Taste**							
**Texture**							

	Dislike very much	Dislike moderately	Dislike slightly	Neither like nor dislike	Like slightly	Like moderately	Like very much
**Color**							
**Size**							
**Smell**							
**Taste**							
**Texture**							

Do you have any comments? Please write them here:

____

____

____

____

____

¡MANY THANKS FOR YOUR PARTICIPATION!
